# Single-center retrospective evaluation of a polymerase chain reaction-based pneumonia panel on antibiotic therapy optimization at a community hospital

**DOI:** 10.1017/ash.2025.57

**Published:** 2025-03-31

**Authors:** Luke Manda, Anthony Wasielewski, Nishika Patel, Timothy P. Gauthier

**Affiliations:** 1 Resident Pharmacist, Baptist Health Boca Raton Regional Hospital, Boca Raton, FL, USA; 2 Clinical Pharmacy Specialist, Infectious Diseases/Antimicrobial Stewardship, Baptist Health Boca Raton Regional Hospital, Boca Raton, FL, USA; 3 Clinical Pharmacy Specialist, Critical Care, Baptist Health Boca Raton Regional Hospital, Boca Raton, FL, USA; 4 Antimicrobial Stewardship Clinical Program Manager, Baptist Health Clinical Pharmacy Enterprise, Miami Lakes, FL, USA

## Abstract

The implementation of a polymerase chain reaction-based pneumonia panel was associated with actionable results in 87% of 384 cases. In a population of mostly elderly non-intensive care unit patients with sputum samples, opportunities for antibiotic stewardship included streamlining for atypical bacteria, *Pseudomonas aeruginosa*, and methicillin-resistant *Staphylococcus aureus* coverage, with occasional opportunities to escalate antibiotic therapy.

## Introduction

The availability of polymerase chain reaction (PCR)-based pneumonia (PN) panels in clinical practice is revolutionary to organism identification speed and has been shown to significantly reduce unnecessary empiric antibiotic use.^1^ Buchan et al reviewed patients with PN panel results, identifying opportunities for antibiotic adjustment in 71% of patients, with antibiotic discontinuation or de-escalation occurring in 48% of patients.^2^ Esplund et al examined the impact of a PN panel on an intensive care unit (ICU) population, finding opportunity for antibiotic changes in 75% of cases, an implementation rate of 54%, and shorter times to antibiotic changes by 29 hours with a 2-day reduction in median antibiotic days of therapy.^3^ Markussen et al evaluated the utilization of a PN panel within an emergency department, finding participants with PN panel results were not only 3.5 times more likely to receive pathogen-directed treatment, but they also received it 9 hours faster than patients without a PN panel.^4^

Indeed, there is a growing body of literature regarding the opportunity of a PN panel to enhance patient care; however, more data are needed to fully elucidate the best use of this emerging technology. The purpose of this study was to evaluate the impact of incorporating a PCR-based PN panel into the management of PN and to identify opportunities to optimize the interpretation and implementation of opportunities created by the panel results.

## Methods

This retrospective, single-center study was conducted at a 400-bed community hospital. In December 2022, the BioFire® FilmArray® PN panel (33 targets, ∼1 hour turnaround)^5^ was introduced as an orderable diagnostic test for acute care respiratory samples. The PN panel results published between December 2022 and September 2023 were extracted from the electronic medical record by laboratory informatics. Patients were screened and excluded if pregnant, incarcerated, found to have positive extrapulmonary cultures, or received systemic antibiotics more than 72 hours prior to the PN panel collection time.

The primary outcome was the incidence of actionable PN panel results. An actionable PN panel result was defined as a result that indicated an opportunity for antibiotic escalation and/or streamlining for PN. PN panel results that failed to identify atypical bacteria, methicillin-resistant *S. aureus* (MRSA), *Pseudomonas aeruginosa* (PSA), or multi-drug resistance gene markers when antibiotics targeting those pathogens were being utilized were designated as streamlining opportunities, as were PN panel results that did not identify any pathogens when antibiotics were being utilized. Secondary outcomes included the implementation of actionable PN panel results, time to implementation, hospital length of stay, all-cause in-hospital mortality, readmission within 30 days of discharge due to any cause, and intervention by the antimicrobial stewardship program pharmacist. Implemented actionable panel results were defined as any modification of the antibiotic regimen that was concordant with the opportunity created by the actionable PN panel. Durations of targeted therapy for MRSA and PSA were evaluated for patients with the respective streamlining opportunity.

Statistical analysis was performed using the Kruskal–Wallis test for continuous variables and either the chi-squared or Fisher’s Exact test for categorical variables. Interquartile ranges from the first quarter to the third quarter were reported for time-dependent variables in addition to the median value. A prespecified two-sided *P* value of <.05 identified statistical significance. The study was approved by the local institutional review board.

## Results

Of 507 patients screened, 384 were included in the analysis. Reasons for exclusion were positive extrapulmonary culture (n = 65) and receipt of antibiotics 72 hours or more before PN panel (n = 58). Actionable results were identified in 333 cases (87%). Baseline characteristics for the entire cohort as well as patients with actionable versus non-actionable results are displayed in Table 1. Concordant PN panel and culture results were found in 361 cases (94%). Of the 23 discordant results, 22 (96%) were PN panels that identified pathogens that did not grow on culture (including 9 related to MRSA and 4 related to PSA). One discordant result was identified for a negative PN panel, but PSA grew on culture. Pharmacist intervention was found for 24 cases (6%).

**Table 1.** Baseline characteristics including data on cases with actionable versus non-actionable results

Characteristic	Overall (n = 384)	Actionable (n = 333)	Non-Actionable (n = 51)	*P* value
Median age, years (IQR)	78.5 (69, 86)	78 (68, 86)	80 (75, 88)	0.262
Race, n (%)				
White	326 (85)	278 (83)	48 (94)	0.142
Black	17 (4)	16 (5)	1 (2)	
Other	41 (11)	39 (12)	2 (4)	
Male gender, n (%)	208 (54)	184 (55)	24 (47)	0.346
Panel collected within 48 h of admission, n (%)	347 (90)	301 (90)	46 (90)	1.000
Severity of illness, n (%)				
ICU care at time of culture collection	35 (9)	29 (9)	5 (10)	0.439
Presence of mechanical ventilation at time of culture collection	15 (4)	13 (4)	2 (4)	0.680
Type of specimen collected, n (%)				
Sputum	353 (92)	309 (93)	44 (86)	0.398
Bronchial washings	7 (2)	5 (2)	2 (4)	
Endotracheal suction	18 (5)	14 (4)	4 (8)	
Nasotracheal suction	6 (2)	5 (2)	1 (2)	
Patient location at time of PN panel order, n (%)				
General floor	269 (70)	233 (70)	36 (71)	0.690
Emergency room	80 (21)	71 (21)	9 (18)	
Intensive care unit	35 (9)	29 (9)	6 (12)	
Ordering provider specialty, n (%)				
Internal medicine	235 (61)	206 (62)	29 (57)	0.895
Pulmonology	92 (24)	78 (23)	14 (27)	
Critical care	30 (8)	25 (8)	5 (10)	
Infectious diseases	21 (5)	19 (6)	2 (4)	
Emergency medicine	6 (2)	5 (2)	1 (2)	
PN panel results by pathogen, n (%)				
No pathogen detected	220 (57)	184 (55)	36 (71)	
*Haemophilus influenzae*	54 (14)	51 (15)	2 (4)	
*Staphylococcus aureus*	46 (12)	45 (14)	1 (2)	
MRSA	26 (7)	26 (8)	0 (0)	
MSSA	20 (5)	19 (6)	1 (2)	
*Pseudomonas aeruginosa*	33 (9)	28 (8)	5 (10)	
Streptococci	26 (7)	23 (7)	0 (0)	
*S. pneumoniae*	12 (3)	12 (4)	1 (2)	
*S. agalactiae*	8 (2)	8 (2)	0 (0)	
*S. pyogenes*	6 (2)	3 (<1)	1 (2)	
*Moraxella catarrhalis*	21 (5)	18 (5)	3 (6)	
*Escherichia coli*	18 (5)	15 (5)	1 (2)	
*Klebsiella*	17 (4)	17 (5)	0 (0)	
*K. pneumoniae*	16 (4)	16 (5)	0 (0)	
*K. aerogenes*	1 (<1)	1 (<1)	0 (0)	
*Serratia marcescens*	7 (2)	6 (2)	1 (2)	
Enterobacter cloacae	5 (1)	4 (1)	1 (2)	
*Acinetobacter* spp.	3 (<1)	2 (<1)	1 (2)	
*Legionella pneumophila*	3 (<1)	3 (<1)	0 (0)	
*Proteus* spp.	1 (<1)	1 (<1)	0 (0)	

Note. IQR, interquartile range; ICU, intensive care unit; PN, pneumonia; MRSA, methicillin-resistant *S. aureus*.

Classification of actionable results and those that were implemented is provided in Table 2. For implemented actionable results, the median time from PN result to antimicrobial regimen modification was 22 hours overall, with a median of 23 hours for streamlining opportunities and 15 hours of escalation opportunities. With respect to the time of the culture result, the opportunities identified by the PN panel results were implemented by a median time of 24 hours before culture results were finalized. Implementation of streamlining and escalation opportunities were implemented in a median time of 22 hours and 45 hours, respectively, before culture results were finalized.

**Table 2.** Classification of actionable pneumonia panel results and those that were implemented

	Actionable cases (n = 333)	Action implemented (n = 218)
Opportunities for antibiotic streamlining	280 of 333 (84)	183 of 218 (84)
Discontinuation of atypical coverage^1^	221 of 280 (79)	123 of 183 (67)
Discontinuation of PSA coverage	138 of 280 (49)	27 of 183 (15)
Discontinuation of MRSA coverage	88 of 280 (31)	50 of 183 (27)
Opportunities for antibiotic escalation	34 of 333 (10)	22 of 218 (10)
Addition of MRSA coverage	16 of 34 (47)	4 of 22 (18)
Addition of PSA coverage	11 of 34 (32)	8 of 22 (36)
Addition of MDR coverage	5 of 34 (15)	3 of 22 (14)
Opportunities for both escalation and streamlining	19 of 333 (6)	13 of 218 (6)
Discontinuation of atypical coverage and addition of PSA coverage	6 of 19 (32)	5 of 13 (38)
Discontinuation of PSA coverage and addition of MRSA coverage	5 of 19 (26)	1 of 13 (8)
Discontinuation of atypical coverage and addition of MRSA coverage	3 of 19 (16)	3 of 13 (23)

Note. PSA, *Pseudomonas aeruginosa*; MRSA, methicillin-resistant *S. aureus*; MDR, multi-drug resistance.

1 Specific streamlining and escalation opportunities were counted per instance, not per patient.

Patients with actionable results that were implemented had a median hospital length of stay of 6 days versus 3 days for those with results not implemented (*P* < 0.01). All-cause mortality was 3% (n = 7) for patients with implemented results compared to 2% (n = 2) for those where it was not implemented (*P* = 0.72). Readmission within 30 days occurred in 29 patients (n = 13%) who had actionable results implemented versus 7 patients (6%) for those where it was not implemented (*P* = 0.19). Comparing results implemented within 24 hours (n = 118) to those implemented after 24 hours (n = 100), the median hospital length of stay was 4 days when implemented within 24 hours versus 6 days when implemented after 24 hours. Comparing patients with interventions implemented versus not implemented, no difference was found in all-cause mortality or readmission rates.

## Discussion

This analysis found that a high percentage of PN panels result in actionable opportunities for antimicrobial stewardship. These actionable results mostly created opportunities for streamlining of atypical bacteria, PSA, and MRSA coverage, with occasional opportunities to escalate antibiotic therapy.

The patient population in this study was predominantly elderly and white, not requiring ICU-level care or mechanical ventilation at the time of PN panel collection. Internal medicine was the most frequent ordering provider type, and sputum specimens were the large majority of collected specimen types, often collected within 48 hours of hospital admission. Population characterization is important, as the lack of clinical benefit found within this study does not necessarily reflect a potential lack of benefit in a particular subgroup. For example, previous studies have demonstrated high sensitivity and specificity of the PN panel with bronchoalveolar lavage samples, while results from this study support the assertion that the PN panel maintains a high level of accuracy and reliability when utilized with sputum samples.^2^

This study is limited by the single-center observational design, dependence on accurate specimen collection, and lack of focused education to prescribers. A key component to introducing new tests is ensuring that actionable opportunities are implemented. Although approximately 9 in 10 tests had an actionable result, only 65% were implemented, and only about half were implemented within 24 hours. Consistent with suggestions from existing literature, incorporation of PN panel results into routine antimicrobial stewardship activities completed by pharmacists (including provider education) will be one next step, while implementing a clinical decision support system will be a second step.^6^ A clinical benefit is more likely to be observed if opportunities are acted upon more frequently and expeditiously.

## Conclusion

Implementation of a PN panel can produce a large amount of actionable results, which can improve antibiotic streamlining and escalation, but implemented opportunities may not globally improve clinical outcomes.
